# Diet-Induced Browning of White Adipose Tissue: Molecular Targets, Mechanisms, and Therapeutic Potential

**DOI:** 10.3390/cimb48020201

**Published:** 2026-02-11

**Authors:** Zhi-Da Yang, Jia-Wei Chen, Ying-Xiu Mei, Xiu-Wen Xia, Yan-Ju Gong, Wei-Jun Ding

**Affiliations:** Department of Fundamental Medicine, Chengdu University of Traditional Chinese Medicine, Chengdu 611137, China

**Keywords:** browning of white adipose tissue, beige adipocytes, dietary bioactive compounds, thermogenesis, AMPK signaling, PPAR pathways, sirtuins, obesity management

## Abstract

Obesity, driven by chronic energy imbalance, has become a major global health burden and is strongly associated with metabolic disorders, including diabetes, hypertension, and cardiovascular disease. Conventional pharmacotherapies often exhibit limited long-term efficacy and are accompanied by undesirable side effects, highlighting the urgent need for safer and more sustainable strategies. Browning of White adipose tissue (WAT)—a process in which white adipocytes acquire brown fat-like thermogenic characteristics—has emerged as a promising approach to enhance energy expenditure and counteract obesity. Increasing evidence demonstrates that various diets and naturally occurring dietary bioactive compounds can effectively induce WAT browning through diverse molecular pathways. Among these, AMPK-, PPAR-, SIRT-, TRP channel-, β3-adrenergic-, and FGF21-related signaling cascades represent the major regulatory hubs linked to mitochondrial biogenesis, lipid metabolism, and thermogenesis. This review summarizes recent advances in diet-induced WAT browning, with particular emphasis on key dietary ingredients, their molecular targets, mechanistic pathways, and metabolic benefits. By integrating findings from in vitro studies, animal models, and emerging translational research, we provide updated insights that may guide the development of novel nutritional interventions, functional foods, and therapeutic strategies for obesity prevention and management.

## 1. Introduction

Obesity has emerged as one of the most significant global health challenges, driven largely by a chronic imbalance between caloric intake and energy expenditure. Excessive lipid accumulation is closely associated with a spectrum of metabolic diseases, including type 2 diabetes, hypertension, cardiovascular disorders, and non-alcoholic fatty liver disease [1,2,3]. Although pharmacological therapies have shown efficacy in promoting weight loss and improving metabolic parameters, their long-term effectiveness is often limited, and adverse effects remain a concern. These limitations highlight the need for alternative and complementary strategies that target fundamental mechanisms of energy balance regulation.

Adipose tissue exists mainly in two functionally distinct forms: white adipose tissue (WAT) and brown adipose tissue (BAT). WAT primarily serves as an energy reservoir, storing triglycerides within large unilocular lipid droplet [4,5]. In contrast, BAT specializes in energy dissipation through non-shivering thermogenesis, a process mediated by high mitochondrial content and elevated expression of uncoupling protein 1 (UCP1) [4]. While metabolically active BAT has been identified in adult humans, its overall abundance is relatively limited and exhibits substantial interindividual variability [6,7], suggesting that strategies relying solely on BAT activation may have restricted applicability at the population level.

In addition to classical brown adipocytes, a distinct population of thermogenically competent adipocytes can emerge within WAT depots in response to external stimuli, including cold exposure, physical exercise, hormonal signals, and nutritional factors [8]. These cells are commonly referred to as beige or “brite” adipocytes. Although the terms “beige” and “brite” originated from different experimental contexts, they are now widely used interchangeably to describe inducible UCP1-positive adipocytes that are developmentally and functionally distinct from classical brown adipocytes. Beige adipocytes exhibit multilocular lipid droplets, high mitochondrial density, and UCP1-dependent thermogenic activity comparable to classical brown adipocytes. Genetic studies further highlight their physiological importance, as mice lacking UCP1 display impaired thermogenesis and are prone to obesity when exposed to thermoneutral conditions [9]. The inducibility of beige adipocytes within WAT makes them a particularly attractive therapeutic target, especially for metabolic disorders driven by excessive energy storage [10,11].

Growing attention has been directed toward the potential of diet and dietary bioactive compounds to induce WAT browning. Compared with pharmacological agents, dietary components offer advantages such as high accessibility, long-term safety, and broad acceptability among the general population. Numerous dietary molecules—including polyphenols, flavonoids, amides, unsaturated fatty acids, and plant-derived phytochemicals—have been shown to trigger beige adipocyte formation or activate thermogenic gene programs. For instance, Sal A, a polyphenolic compound, has been shown to increase UCP1 levels in white adipose tissue and cultured adipocytes [12]. However, it is important to recognize that many experimental studies employ doses exceeding typical dietary intake, and the observed effects should primarily be interpreted in a mechanistic context.

**Figure 1:**
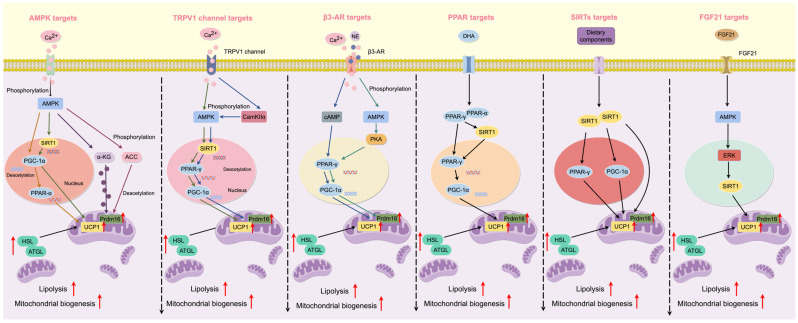
Graphical summary of the principal molecular targets and signaling pathways mediating diet-induced browning of white adipose tissue (WAT). Dietary bioactive compounds promote WAT browning through multiple convergent mechanisms, including activation of AMP-activated protein kinase (AMPK), peroxisome proliferator-activated receptor γ (PPARγ), transient receptor potential (TRP) channels (e.g., TRPV1), β3-adrenergic receptors (β3-AR), sirtuin-mediated mitochondrial regulation (SIRTs), and fibroblast growth factor 21 (FGF21) signaling. These pathways collectively induce mitochondrial biogenesis, thermogenic gene expression, and beige adipocyte recruitment. Figure created using FigDraw (version 2.0) (ID: AWWIUdadc4) and reproduced with permission.

The present article is a narrative mechanistic review rather than a formal systematic review. By integrating evidence from in vitro studies, animal models, and emerging human research, we aim to synthesize current knowledge on how diets and dietary bioactive compounds influence WAT browning through convergent molecular pathways. Emphasis is placed on identifying key signaling networks—such as AMPK, PPARγ, β-adrenergic signaling, TRP channels, sirtuin-mediated mitochondrial regulation, and FGF21 signaling—that collectively govern thermogenic activation. An overview schematic summarizing these pathways is provided (Figure 1) to serve as a conceptual framework for the subsequent sections. Finally, given that physical exercise is a well-established inducer of adipose tissue browning and mitochondrial biogenesis, potential interactions and overlapping mechanisms between dietary factors and exercise are briefly considered and discussed in later sections.

## 2. The Critical Role of Beige Adipocytes in Energy Metabolism

Beige adipocytes have emerged as key regulators of systemic energy homeostasis due to their ability to dissipate chemical energy as heat. Unlike classical white adipocytes, which primarily function as long-term energy storage depots, beige adipocytes possess a thermogenic phenotype characterized by multilocular lipid droplets, abundant mitochondria, and high expression of uncoupling protein 1 (UCP1). These features enable beige adipocytes to engage in adaptive thermogenesis, thereby increasing whole-body energy expenditure and improving metabolic flexibility [13,14,15].

Mitochondrial oxidative metabolism is central to the thermogenic function of beige adipocytes. Upon activation, beige adipocytes markedly increase glucose uptake and fatty acid β-oxidation to fuel the tricarboxylic acid (TCA) cycle and sustain elevated respiratory activity [13]. UCP1, located in the inner mitochondrial membrane, uncouples oxidative phosphorylation by dissipating the proton gradient as heat rather than conserving energy as ATP. Importantly, this UCP1-dependent mechanism is indispensable for adaptive thermogenesis, as other uncoupling protein homologs such as UCP2 and UCP3 do not compensate for the loss of UCP1 in mediating cold- or diet-induced thermogenic responses [14,15]. Accordingly, UCP1 has been widely recognized as a central molecular effector linking beige adipocyte activation to increased energy expenditure and protection against obesity.

The inducible nature of beige adipocytes further underscores their physiological and therapeutic relevance. In contrast to classical BAT, which is anatomically restricted and tends to decline with age in humans, beige adipocytes can be recruited de novo within white adipose tissue depots in response to a variety of external stimuli. Environmental factors such as cold exposure and physical activity, endocrine signals including catecholamines and thyroid hormones, as well as nutritional factors derived from the diet, all contribute to the differentiation and activation of beige adipocytes. This plasticity enables white adipose tissue to function as an adaptable energy-dissipating organ that can be modulated by lifestyle-related interventions.

Accumulating evidence from animal models indicates that dietary compounds represent particularly effective modulators of beige adipocyte recruitment and function. For instance, emodin, a natural anthraquinone derived from *Rheum palmatum*, increases the expression of beige adipocyte markers such as CD137 in subcutaneous WAT of diet-induced obese (DIO) mice [16]. Pentamethylquercetin (PMQ), a polymethoxyflavone, enhances the expression of the mitochondrial biogenesis regulator PGC-1α in a dose-dependent manner, partly through stimulating irisin secretion in skeletal muscle—a myokine markedly reduced in individuals with obesity [17,18]. Similarly, hydroxy-α-sanshool (HAS), an amide compound derived from *Zanthoxylum* species, promotes WAT browning and elevates energy expenditure, demonstrating its potential as a dietary metabolic regulator [19]. Comparable browning-inducing effects have been reported for a broad range of dietary phytochemicals and nutrients, underscoring the general relevance of nutrition-derived signals in beige adipocyte biology [20,21,22].

Although most mechanistic insights into beige adipocyte function are derived from cell-based and rodent studies, emerging evidence indicates that inducible thermogenic adipocytes also exist in adult humans, albeit with distinct patterns of regulation compared with rodents [23,24,25]. Human adipose tissue depots exhibit heterogeneity in browning responses, and the magnitude of beige marker expression and thermogenic activity varies across depots and between sexes, potentially reflecting differences in hormonal regulation and intrinsic adipocyte precursor populations [26,27]. In addition, although physical exercise robustly stimulates thermogenic signaling in animals, evidence for exercise-induced beiging in human white adipose tissue remains mixed, underscoring species differences and the complexity of physiological regulation in humans [28,29]. Moreover, several signaling pathways involved in beige adipocyte activation, such as sympathetic nervous system–mediated β-adrenergic signaling and mitochondrial biogenesis pathways, overlap with those induced by physical exercise, suggesting that beige adipocytes may operate within an integrated physiological network responding to both nutritional and physical stimuli rather than as isolated thermogenic units [30].

Collectively, beige adipocytes represent a metabolically active and inducible adipocyte population with significant implications for energy balance and metabolic disease. Their ability to convert stored energy into heat, together with their responsiveness to dietary, hormonal, and environmental cues, positions them as an attractive therapeutic target for obesity and related metabolic disorders. A detailed understanding of how dietary components modulate the biogenesis and functional activity of beige adipocytes is, therefore, essential for the development of effective, nutrition-based strategies to improve metabolic health.

## 3. The Effects and Mechanisms of Diet and Dietary Components in WAT Browning

Dietary factors have attracted growing interest as modulators of white adipose tissue (WAT) browning because of their capacity to influence systemic energy metabolism through multiple converging mechanisms. A growing body of experimental evidence indicates that numerous dietary components—including polyphenols, flavonoids, and amide compounds—can actively contribute to the induction of WAT browning. Beyond isolated bioactive molecules, several commonly consumed foods and beverages have also been reported to exhibit browning-promoting effects in preclinical models. For example, long-term, low-dose alcohol intake has been shown to elevate UCP1 expression in adipose tissue, thereby attenuating the decline in energy expenditure typically observed during high-fat diet feeding [31]. In addition, gastric infusion of green tea in rats upregulated the expression of beige adipocyte biomarkers in WAT [32], and similar browning-related effects have been reported across multiple dietary intervention studies [33,34,35].

Importantly, diet-induced WAT browning is not limited to the upregulation of UCP1 alone. Other thermogenic and beige adipocyte–associated markers, including PR domain containing 16 (Prdm16), peroxisome proliferator-activated receptor gamma coactivator 1-alpha (PGC-1α), cell death-inducing DFFA-like effector a (Cidea), CD137, and transmembrane protein 26 (Tmem26), are frequently induced by dietary components, reflecting the involvement of complex and coordinated regulatory networks in beige adipocyte biogenesis and activation.

The studies summarized in this section encompass diverse experimental models, including cell-based systems, rodent studies, and a limited but expanding body of human research. In vitro experiments primarily provide mechanistic insights into signaling pathways regulating beige adipocyte differentiation and activation, whereas animal studies allow integrated evaluation of thermogenesis and metabolic outcomes under controlled dietary conditions. Human studies, although fewer and more heterogeneous in design, offer essential translational perspectives while also revealing substantial interindividual variability in browning responsiveness.

The rigor and strength of the available evidence vary across studies. Most mechanistic conclusions are supported by well-controlled animal experiments using defined dietary compounds and doses, whereas human investigations often rely on indirect markers of browning or short-term interventions. Differences in species, adipose depot, sex, age, and metabolic status further contribute to variability in reported outcomes. Accordingly, this review integrates findings across experimental levels to identify conserved signaling pathways while acknowledging limitations in translational extrapolation.

An additional consideration is the interaction between dietary interventions and physical activity. Physical exercise is a well-established stimulus for mitochondrial biogenesis and metabolic remodeling in adipose tissue and engages several pathways implicated in diet-induced browning, including sympathetic activation and AMP-activated protein kinase (AMPK) signaling. Consequently, separating the independent contributions of diet and exercise—particularly in human studies—can be challenging. Where applicable, this review specifies whether dietary compounds were administered in isolation or alongside exercise-related stimuli.

In the following subsections, dietary components are organized according to their principal molecular targets and signaling pathways involved in WAT browning, including AMPK, peroxisome proliferator-activated receptor γ (PPARγ), transient receptor potential (TRP) channels, β3-adrenergic receptors (β3-AR), sirtuins (SIRTs), fibroblast growth factor 21 (FGF21), and other emerging mechanisms (Figure 1).

### 3.1. AMPK

AMP-activated protein kinase (AMPK) is a central cellular energy sensor that orchestrates metabolic adaptation to energy stress by promoting ATP-generating catabolic pathways while suppressing energy-consuming anabolic processes. Owing to its pivotal role in regulating mitochondrial biogenesis, lipid oxidation, and thermogenesis, AMPK has emerged as one of the most extensively studied molecular targets in diet-induced WAT browning [36].

A substantial body of evidence indicates that numerous dietary bioactive compounds induce WAT browning through AMPK-dependent signaling pathways (Table 1). A well-characterized mechanism involves the AMPK-PGC1-α/SIRT1 pathway, which coordinates mitochondrial remodeling and thermogenic gene expression. For example, ginger extract activates AMPK, leading to upregulation of SIRT1 and subsequent deacetylation and activation of PGC-1α. Activated PGC-1α promotes the expression of thermogenic regulators, including PRDM16 and UCP1, thereby enhancing mitochondrial content and beige adipocyte formation [37]. This pathway illustrates how AMPK integrates dietary signals with transcriptional programs governing thermogenesis.

Additional studies further support AMPK as an upstream initiator of thermogenic programming. Salvianolic acid A (Sal A)–induced browning is markedly attenuated by pharmacological or genetic inhibition of AMPK, whereas silencing SIRT1 does not affect AMPK phosphorylation, indicating a unidirectional regulatory hierarchy in which AMPK functions upstream of SIRT1 [12]. These findings reinforce the role of AMPK as a primary trigger of downstream thermogenic signaling in response to dietary compounds.

Beyond the canonical AMPK–SIRT1–PGC-1α pathway, AMPK regulates WAT browning through alternative downstream targets. Secoisolariciresinol diglucoside (SDG), a lignan derived from flaxseed, promotes beige adipocyte activation via an AMPK–acetyl-CoA carboxylase (ACC) signaling cascade, as demonstrated by loss-of-function studies using AMPKα inhibition or siRNA knockdown [38]. Similarly, L-theanine, a major bioactive component of green tea, induces browning through an AMPK/α-ketoglutarate/PRDM16 axis, underscoring the diversity of metabolic routes through which AMPK governs adipose tissue remodeling [40].

Collectively, these findings establish AMPK as a central metabolic hub linking dietary cues to adipocyte thermogenic programming. By coordinating nutrient-derived signals with mitochondrial adaptations and transcriptional control of thermogenic genes, AMPK activation represents a promising mechanistic basis for nutritional strategies aimed at enhancing WAT browning and energy expenditure. However, as AMPK is also robustly activated by physical exercise, careful consideration of dose, experimental context, and coexisting lifestyle factors is essential when evaluating the translational relevance of diet-induced AMPK activation.

### 3.2. PPARγ

Peroxisome proliferator-activated receptor γ (PPARγ) is a nuclear receptor that plays a central role in adipocyte differentiation, lipid metabolism, and adipose tissue remodeling (Table 2). Although PPARγ is classically recognized as a master regulator of adipogenesis, accumulating evidence indicates that under specific regulatory contexts, PPARγ also contributes to white adipose tissue (WAT) browning by coordinating transcriptional programs associated with mitochondrial biogenesis and thermogenesis.

A central mechanism underlying PPARγ-mediated browning involves its interaction with thermogenic coactivators, particularly PGC-1α and PRDM16. PPARγ activation enhances the transcription of PGC-1α, a master regulator of mitochondrial biogenesis and oxidative metabolism. In turn, PGC-1α forms a transcriptional complex with PPARγ, reinforcing the expression of thermogenic genes such as UCP1 and promoting the acquisition of beige adipocyte characteristics [32,48,60]. This cooperative transcriptional network enables PPARγ to support thermogenic programming beyond its canonical adipogenic function.

Post-translational regulation further refines the browning-related activity of PPARγ. Deacetylation of PPARγ has been identified as a critical determinant that favors thermogenic gene expression while limiting excessive lipid accumulation. SIRT1, a key NAD^+^-dependent deacetylase, interacts with PPARγ to modulate its acetylation status, thereby facilitating PRDM16 recruitment and enhancing beige adipocyte gene programs [66]. These findings highlight that PPARγ-driven browning is highly dependent on its regulatory context rather than simple receptor activation.

Dietary components have been shown to modulate PPARγ signaling and contribute to WAT browning through these mechanisms. Omega-3 fatty acids, such as docosahexaenoic acid (DHA), as well as nutrients rich in chia oil, upregulate PPARγ expression and are associated with enhanced thermogenic gene expression and improved metabolic parameters in animal models [57,58]. Importantly, these dietary lipids appear to fine-tune PPARγ transcriptional activity toward thermogenic outcomes, distinguishing their effects from those of full synthetic PPARγ agonists that often promote adipogenesis.

In addition to PPARγ, other PPAR family members, particularly PPARα, also participate in adipose tissue remodeling and thermogenic regulation [67]. PPARα activation is closely linked to enhanced lipid oxidation and mitochondrial metabolism. Long-term low-concentration alcohol intake has been reported to induce UCP1 expression in a PPARα-dependent manner [31], and dietary citrate upregulates PPARα expression in obese mice, promoting lipid catabolism and browning-related gene expression [33]. Together, these observations underscore the coordinated roles of PPAR isoforms in governing adipose thermogenic plasticity [68,69].

Altogether, PPARγ functions as a context-dependent transcriptional hub linking dietary cues to beige adipocyte development. Through dynamic interactions with PGC-1α, PRDM16, SIRT1, and other PPAR family members, PPARγ integrates nutritional signals into thermogenic transcriptional programs. Dietary modulation of this pathway, therefore, represents a nuanced strategy to promote WAT browning, provided that receptor activation is tightly regulated to balance thermogenesis and adipogenesis.

### 3.3. TRP Channels

Transient receptor potential (TRP) channels constitute a large family of cation-permeable membrane proteins that function as polymodal sensors of thermal, chemical, and mechanical stimuli. Several TRP subfamilies—including TRPV, TRPA, and TRPM—have been implicated in metabolic regulation and the induction of white adipose tissue (WAT) browning. These channels are expressed not only in sensory neurons but also in adipocytes and adipose-resident cells, enabling dietary stimuli to directly or indirectly modulate thermogenic programming (Table 3).

Among TRP channels, TRPV1 is the most extensively studied in diet-induced browning, particularly in diet-induced obese (DIO) models. Activation of TRPV1 by dietary bioactive compounds frequently triggers intracellular Ca^2+^ influx, leading to activation of AMPK-dependent signaling cascades and downstream thermogenic transcriptional programs. Hydroxy-α-sanshool (HAS), a bioactive amide derived from *Zanthoxylum bungeanum* fruit, robustly upregulates UCP1 expression both in vitro and in vivo via TRPV1 activation. Mechanistically, HAS-induced browning requires SIRT1-dependent deacetylation of PPARγ, thereby linking TRPV1 activation to the AMPK–SIRT1–PPARγ axis and beige adipocyte gene expression [19]. These findings position TRPV1 as a molecular interface connecting dietary sensory cues to adipocyte thermogenic remodeling.

Capsaicin, a prototypical TRPV1 agonist derived from *Capsicum annuum*, is among the most widely investigated dietary thermogenic agents. Capsaicin activates AMPK and SIRT1, promotes deacetylation of thermogenic transcription factors, and induces UCP1 expression in WAT [70]. Importantly, the browning effect of capsaicin is abolished in TRPV1-deficient mice, confirming the essential role of TRPV1 signaling. Notably, dose-dependent effects have been reported: low concentrations of capsaicin promote beige adipocyte formation through TRPV1-dependent mechanisms, whereas higher concentrations inhibit adipogenesis through TRPV1-independent pathways [73]. This biphasic response underscores the complexity of TRP-mediated metabolic regulation and highlights the importance of dosage and exposure context.

TRPA1 represents another TRP subtype involved in diet-induced thermogenesis. 10-Hydroxy-trans-2-decenoic acid (HDEA), a natural TRPA1 agonist present in royal jelly, enhances thermogenesis in white adipocytes by stimulating β-adrenergic receptor signaling [75]. This TRPA1–β-adrenergic interaction illustrates the convergence of sensory channel activation with classical sympathetic pathways governing adipose browning.

Cold-sensitive TRPM8 channels also contribute to energy homeostasis and thermogenic regulation. TRPM8 deficiency results in increased heat loss, elevated food intake, and impaired thermoregulation, ultimately predisposing mice to obesity [76]. Conversely, activation of TRPM8 by dietary menthol or synthetic agonists induces WAT browning and attenuates weight gain in DIO mice [72,77], suggesting that TRPM8 serves as a sensor coupling cooling-related dietary or environmental cues to thermogenic responses.

Collectively, TRP channels function as molecular conduits that translate dietary chemical and thermal signals into metabolic responses promoting WAT browning. Through Ca^2+^-dependent signaling, AMPK activation, and engagement of β-adrenergic pathways, TRPV1, TRPA1, and TRPM8 integrate nutritional and sensory inputs into thermogenic transcriptional networks. Although most evidence derives from cell-based and animal studies, these pathways overlap substantially with established mechanisms of exercise- and cold-induced browning, underscoring the physiological relevance of TRP channels as targets for dietary modulation of energy expenditure.

### 3.4. β3-AR

The β3-adrenergic receptor (β3-AR) is a key component of the sympathetic nervous system and plays a critical role in regulating thermogenesis in both brown and white adipose tissues. β3-ARs are primarily expressed on the surface of adipocytes, where it mediates catecholamine-induced lipolysis, mitochondrial activation, and the development of beige adipocytes [78,79]. Activation of β3-AR promotes glucose uptake, enhances fatty acid mobilization, and triggers transcriptional programs that favor adaptive thermogenesis [80,81]. As such, β3-AR represents a central node through which dietary factors, environmental stimuli, and neural inputs converge to regulate adipose tissue remodeling.

A growing number of dietary bioactive compounds have been reported to induce white adipose tissue (WAT) browning through β3-AR–dependent mechanisms. Trans-cinnamic acid (tCA), a natural constituent of cinnamon, induces a beige-like phenotype in 3T3-L1 adipocytes by activating β3-AR and promoting thermogenic gene expression [82]. Similarly, D-mannitol, a sugar alcohol commonly used in food processing, has been shown to stimulate β3-AR signaling, enhance protein kinase A (PKA) activation, and promote browning of WAT [83]. Plant-derived compounds such as curcumin and lotus leaf extracts further support this paradigm, as they enhance thermogenic gene expression through β-adrenergic–related pathways [84,85]. Together, these findings highlight the capacity of diverse dietary constituents to modulate adipose thermogenesis via β3-AR signaling.

Pharmacological evidence provides additional support for the central role of β3-AR in thermogenic activation. Mirabegron, a selective β3-AR agonist clinically approved for the treatment of overactive bladder, robustly increases UCP1 expression and enhances brown or beige adipose tissue activity in both rodents and humans [86]. These observations underscore the physiological relevance of β3-AR signaling in humans, while also highlighting species-specific differences in receptor expression, adipose depot responsiveness, and translational efficacy.

Mechanistically, β3-AR signaling is closely integrated with upstream sensory and metabolic pathways. Activation of transient receptor potential (TRP) channels by dietary agonists can stimulate afferent sensory signaling to the central nervous system, leading to sympathetic nerve activation and norepinephrine release. Norepinephrine subsequently binds β3-AR on adipocytes, triggering cAMP-PKA signaling cascades that culminate in UCP1 induction and beige adipocyte recruitment [71]. This TRP–sympathetic–β3-AR axis provides a mechanistic framework explaining how dietary compounds indirectly engage adrenergic pathways to promote WAT browning(Table 4).

Collectively, β3-AR functions as a pivotal integrator linking dietary cues, sympathetic activation, and adipocyte thermogenic programming. Through coordination of lipolysis, mitochondrial remodeling, and thermogenic gene expression, β3-AR represents a critical target through which nutritional and lifestyle interventions may enhance energy expenditure and improve metabolic health.

### 3.5. SIRTs

Sirtuins (SIRTs) are a family of NAD^+^-dependent deacetylases that play essential roles in metabolic regulation, mitochondrial function, and cellular adaptation to energy stress. Among the seven mammalian sirtuin isoforms, SIRT1 and SIRT3 have been most extensively investigated in the context of adipose tissue thermogenesis and white adipose tissue (WAT) browning [87] (Table 5). These two sirtuins exert complementary effects on beige adipocyte formation through their distinct subcellular localizations and regulatory targets.

SIRT1 primarily resides in the nucleus but can shuttle to the cytoplasm under specific physiological conditions [93,94]. In adipocytes, SIRT1 promotes WAT browning primarily through transcriptional regulation by deacetylating key thermogenic regulators such as PGC-1α and PPARγ. Deacetylation of PGC-1α enhances its transcriptional activity, thereby promoting mitochondrial biogenesis and thermogenic gene expression. Similarly, SIRT1-mediated deacetylation of PPARγ facilitates the recruitment of PRDM16, shifting PPARγ activity away from lipid storage toward beige adipocyte differentiation. These mechanisms establish SIRT1 as a central transcriptional regulator linking dietary compound–induced metabolic cues to thermogenic programming in adipose tissue [95].

In contrast, SIRT3 is localized predominantly within mitochondria, where it regulates the acetylation status of mitochondrial proteins and is indispensable for maintaining oxidative metabolism and mitochondrial integrity [96,97]. SIRT3 deficiency impairs mitochondrial function and predisposes organisms to metabolic dysfunction, underscoring its essential role in energy homeostasis. Although SIRT3 exerts systemic metabolic effects across multiple tissues—for example, pancreatic SIRT3 deficiency promotes hepatic steatosis by increasing serotonin synthesis in obese mice [98]—its mitochondrial regulatory function is also critical for supporting the elevated oxidative capacity required during adipose thermogenesis. Dietary compounds such as guttiferone J, a bioactive component of *Garcinia cambogia*, activate SIRT3, reduce PGC-1α acetylation, enhance mitochondrial biogenesis, and increase UCP1 expression in adipocytes [88], highlighting SIRT3 as a key mitochondrial effector contributing to diet-induced browning.

Several dietary phytochemicals promote WAT browning through activation of the SIRT1/PGC-1α axis. Sulforaphane, a natural isothiocyanate found in cruciferous vegetables, increases mitochondrial number and respiratory enzyme activity by upregulating the Nrf2/SIRT1/PGC-1α signaling pathway, thereby enhancing thermogenesis in 3T3-L1 adipocytes [89]. Similarly, epicatechin, a flavonoid abundant in green tea, stimulates mitochondrial biogenesis, improves mitochondrial ultrastructure, enhances fatty acid oxidation, and induces WAT browning in obese mice via SIRT1-associated mechanisms [90]. These findings support a role for dietary modulation of sirtuin signaling in shaping adipose thermogenic plasticity.

Collectively, SIRT1 and SIRT3 function as complementary regulators of WAT browning, acting at the transcriptional and mitochondrial levels, respectively. Through coordinated control of transcription factor acetylation, mitochondrial biogenesis, and oxidative metabolism, sirtuins link dietary bioactive compounds to adaptive thermogenesis. While most evidence is derived from cell-based and animal studies, targeting sirtuin signaling through nutritional strategies represents a promising, context-dependent approach to enhancing energy expenditure and improving metabolic health.

### 3.6. FGF21

Fibroblast growth factor 21 (FGF21) is an endocrine hormone predominantly synthesized in the liver, but also expressed in adipose tissue, skeletal muscle, and other metabolic organs. FGF21 plays an important regulatory role in energy homeostasis, glucose and lipid metabolism, and adaptive responses to metabolic stress. Accumulating evidence from animal studies highlights FGF21 as a key hormonal modulator involved in white adipose tissue (WAT) browning and diet-associated metabolic adaptations [99].

FGF21 expression is strongly induced under conditions of metabolic challenge, including fasting, ketogenic diets, and cold exposure. In the context of thermogenesis, FGF21 is upregulated in brown adipose tissue (BAT) following cold stimulation and functions downstream of β-adrenergic signaling [100,101]. Experimental studies demonstrate that FGF21-deficient mice exhibit impaired thermogenic capacity and reduced cold tolerance, whereas exogenous FGF21 administration enhances chronic adaptive thermogenesis, increases energy expenditure, and improves metabolic flexibility [49]. These findings support an essential role for FGF21 in facilitating optimal thermogenic responses and beige adipocyte recruitment in rodents.

Dietary interventions have also been shown to modulate circulating or tissue-specific FGF21 levels, thereby indirectly promoting WAT browning. For instance, supplementation with apple polyphenols upregulates thermogenic gene expression, including *Ucp1*, *Cidea*, and *Cd137*, in WAT via a catecholamine–FGF21–PGC-1α signaling cascade, ultimately facilitating beige adipocyte formation [102] (Table 6). This mechanism illustrates how certain dietary phytochemicals may engage FGF21 as part of a broader neuroendocrine network rather than acting through direct adipocyte-autonomous pathways.

Amino acids, particularly branched-chain amino acids (BCAAs), have also been implicated in the regulation of adipose thermogenesis through FGF21-related signaling. Supplementation with leucine or isoleucine has been reported to activate β3-adrenergic signaling and promote browning via an FGF21–SIRT1–PRDM16 axis [105]. However, subsequent studies suggest that leucine is the primary contributor to this effect, whereas isoleucine may play a more limited or indirect role [103]. These findings underscore the complexity of amino acid–FGF21 interactions and highlight the need for further mechanistic clarification.

Despite robust evidence in animal models, the translational relevance of FGF21-mediated browning in humans remains more nuanced. Circulating FGF21 levels are often elevated in obesity and metabolic syndrome, a phenomenon frequently interpreted as reflecting a state of FGF21 resistance. Clinical studies using pharmacological FGF21 analogs have demonstrated improvements in lipid metabolism and insulin sensitivity, but consistent induction of adipose browning or sustained increases in energy expenditure in humans have been less evident. These observations suggest that FGF21 primarily functions as a metabolic modulator whose thermogenic efficacy is highly context-dependent.

Collectively, FGF21 represents a hormonally mediated regulatory layer linking dietary signals, sympathetic activation, and adipose tissue remodeling. Rather than acting as a sole driver of WAT browning, FGF21 appears to amplify and coordinate thermogenic responses within an integrated metabolic network. Targeting the FGF21 axis through nutritional strategies may therefore contribute to metabolic improvement, provided that tissue specificity, metabolic state, and signaling sensitivity are carefully considered.

### 3.7. Other Targets or Mechanisms

**Figure 2:**
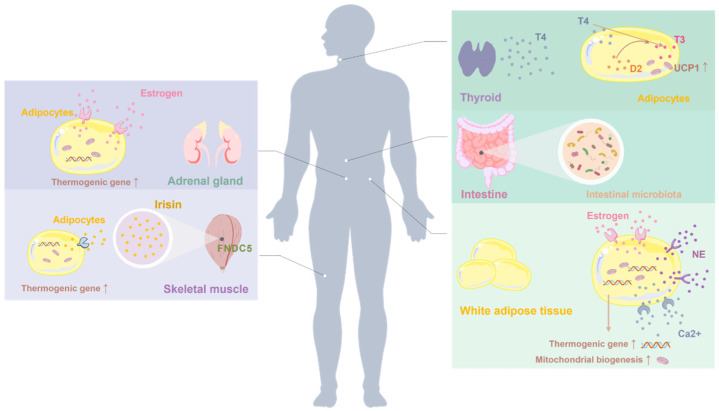
Additional molecular and endocrine targets implicated in the regulation of white adipose tissue (WAT) browning. Beyond canonical thermogenic pathways, emerging regulators such as estrogen, thyroid hormones, and muscle-derived myokines (e.g., irisin) have been shown to influence beige adipocyte recruitment and thermogenic capacity. These factors act through their respective receptors expressed in adipose tissue and interact with metabolic and endocrine signaling networks to modulate WAT plasticity, particularly under conditions of obesity and metabolic stress.

Beyond the core signaling pathways discussed above—including AMPK, PPARs, TRP channels, β3-adrenergic receptors, sirtuins, and FGF21—several additional molecular and physiological mechanisms have been implicated in the dietary regulation of white adipose tissue (WAT) browning. Compared with canonical thermogenic pathways, these mechanisms are less extensively characterized and are generally considered modulatory or context-dependent. Nevertheless, they contribute to the broader regulatory landscape of adipocyte thermogenic remodeling (Figure 2).

One such mechanism involves cyclic guanosine monophosphate (cGMP) signaling. Elevation of intracellular cGMP levels activates protein kinase G (PKG), which in turn induces the expression of thermogenic regulators such as PGC-1α and UCP1 [106]. This suggests that dietary components capable of modulating nitric oxide production or influencing guanylate cyclase activity may promote WAT browning through cGMP-dependent mechanisms.

The phosphoinositide 3-kinase (PI3K)–Akt–mTOR axis has also been implicated in diet-induced browning. Apigenin, a flavonoid present in various plant-based foods, has been reported to activate this pathway, leading to enhanced mitochondrial biogenesis and increased thermogenic gene expression in adipose tissue [107]. These findings highlight the complexity of nutrient–signal integration in adipocytes, while also suggesting that PI3K–Akt–mTOR signaling may function as a permissive or supportive pathway rather than a primary driver of browning.

Endocrine regulation represents another important layer of control. Thyroid hormones, particularly triiodothyronine (T3), are well-established regulators of basal metabolism and thermogenesis and have been increasingly linked to beige adipocyte recruitment. Elevated T3 levels enhance oxidative metabolism and upregulate UCP1 expression, thereby amplifying thermogenic responses to dietary or environmental stimuli [108]. This illustrates how systemic hormonal cues can interact with nutritional signals to shape adipose tissue thermogenic capacity.

In addition, emerging evidence points to the involvement of sex hormones, gut microbiota, and inter-organ communication in modulating WAT browning. Estrogen signaling enhances mitochondrial function and thermogenic gene expression, providing a mechanistic basis for sex-specific differences in adipose tissue biology [109]. Gut microbiota–derived metabolites, including short-chain fatty acids, influence host energy expenditure and may indirectly facilitate beige adipocyte recruitment [110]. Furthermore, skeletal muscle–derived myokines such as irisin and meteorin-like have been shown to promote WAT browning, suggesting potential synergy between dietary interventions and physical activity [111].

Overall, these additional mechanisms underscore the multifactorial and integrative nature of diet-induced WAT browning. Rather than acting as independent thermogenic pathways, they interact with core signaling networks to modulate the magnitude, tissue specificity, and sustainability of beige adipocyte formation. Further studies are required to clarify their hierarchical relationships and translational relevance in the context of nutritional strategies for obesity and metabolic disease management.

## 4. Discussion

Diet-induced browning of white adipose tissue (WAT) has emerged as a promising strategy to enhance energy expenditure and counteract obesity and related metabolic disorders. This review integrates evidence demonstrating that diverse dietary components—including polyphenols, flavonoids, amides, unsaturated fatty acids, and amino acid–derived metabolites—can promote WAT browning through the coordinated activation of multiple thermogenic pathways, notably AMPK, PPARs, TRP channels, β3-adrenergic receptors, sirtuins, and FGF21. Rather than acting through isolated mechanisms, these dietary bioactives appear to converge on interconnected signaling networks governing mitochondrial biogenesis, lipid oxidation, and beige adipocyte differentiation.

Among these pathways, AMPK and PPARγ represent the most extensively characterized and evolutionarily conserved mediators of diet-induced browning. AMPK functions as a central metabolic sensor integrating energetic cues and coordinating downstream processes such as SIRT1 activation, PGC-1α–driven mitochondrial biogenesis, and lipid oxidation [112]. SIRT1 further fine-tunes thermogenic transcriptional programs by deacetylating PGC-1α and PPARγ, thereby facilitating beige adipocyte recruitment and adaptive thermogenesis [113,114]. Together, these interlinked signaling axes provide a coherent mechanistic framework explaining how nutritional signals are translated into enhanced energy expenditure. Nevertheless, it should be emphasized that the majority of mechanistic evidence is derived from cell-based systems and rodent models, and whether comparable pathway activation occurs in humans at physiologically achievable dietary exposures remains insufficiently defined.

Despite encouraging preclinical findings, several challenges complicate the translation of diet-induced browning into effective human interventions. Many dietary bioactives, such as quercetin or curcumin, exhibit limited bioavailability and extensive metabolic transformation, raising uncertainty as to whether their in vivo thermogenic effects reflect direct adipocyte actions or indirect systemic mechanisms [115]. In addition, substantial heterogeneity exists in beige adipocyte inducibility across adipose depots and individuals, influenced by age, sex hormones, genetic background, environmental temperature, gut microbiota composition, and habitual dietary patterns [116]. These variables likely contribute to the inconsistent browning responses observed in human studies and underscore the necessity of personalized and context-dependent approaches.

Taken together, diet-based strategies to promote WAT browning offer clear advantages over pharmacological interventions, including improved safety profiles and suitability for long-term metabolic management [117]. However, advancing this field will require rigorously designed human trials, improved understanding of in vivo pharmacokinetics, and integrative multi-omic analyses to define which dietary components exert meaningful browning effects, under what conditions, and in which populations. Future research should prioritize translational and personalized frameworks, as well as the potential synergy between dietary bioactives and lifestyle interventions such as exercise, cold exposure, or intermittent fasting [116,118].

## Figures and Tables

**Table 1:** Promotion of WAT browning via AMPK pathway by dietary components.

Compound Name	Source	Experimental Model	Mechanisms	Efficacy	Reference
Ginger	Ginger	DIO mice	Activated SIRT1/AMPK/PG-C-1α pathway	↑ UCP1, Cidea, Prdm16, Tmem26, Cited1, PGC-1α, SIRT1	[37]
Secoisolariciresin-ol diglucoside	Flaxseed	DIO mice, db/db mice, 3T3-L1 cells	Increased AMPKα pathway	↑ UCP1, PGC-1α, Prdm16, AMPKα	[38]
Milk fat globule membrane	Breast milk	DIO rats	Upregulated the phosphorylation of AMPK	↑ UCP1, Prdm16, PGC-1α	[39]
Salvianolic acid A	*Radix Salvia miltiorrhiza*	DIO mice, C3H10T1/2 adipocytes	Activated AMPK-SIRT1 pathway	↑ UCP1, Prdm16, PGC-1α, AMPK, SIRT1, Cidea, Fgf21	[12]
L-Theanine	Green Tea	DIO mice, C3H10T1/2 cells	Activated AMPK/a-KG/Prdm16 axis	↑ UCP1, Prdm16, PGC-1α, Dio2, Cpt1b	[40]
Pentamethylquerc-etin	Plants and fruits	C2C12 cells, 3T3-L1 cells	Activated the AMPK/PGC-1α/FNDC5 signaling pathway	↑ UCP1, PGC1α, Tmem26	[17]
2′-Fucosyllactose	Milk	C3H10T1/2 cells, 3T3-L1 cells	Dependent on the AMPK pathway	↑ UCP1, Prdm16, PGC1α, Cidea, CD137, Tmem26	[41]
Quercetin	Onion	DIO mice, 3T3-L1 cells	Activated AMPK α1/SIRT1	↑ UCP1	[42]
Mangiferin	*Mangifera indica*	3T3-L1 cells	Possibly activated AMPK signaling	↑ UCP1, PGC-1α, Prdm16, PPAR-α, PPAR-γ, CIDEA, DIO2, SIRT1, NRF1	[43]
6-Gingerol	Ginger	3T3-L1 cells	Activated the AMPK pathway	↑ UCP1, PGC-1α, Prdm16, Cited1, SIRT1, Tmem26	[44]
Cordycepin	Cordycepsc	DIO mice, 3T3-L1 cells	Partly through induction of AMPK activation	↑ UCP1	[45]
Bergamottin	Grapefruit juice	DIO mice, 3T3-L1 cells	Activated the AMPK pathway	↑ UCP1, PGC-1α, p-AMPKα	[46]
Naringenin	*Citrus reticulata*	Human white adipocyte	Chronic AMPK activation	↑ UCP1, PGC-1α, PGC-1β	[47]
*Panax ginseng* and *Diospyros kaki* leaf	*Panax ginseng* and * Diospyros kaki*	DIO mice, 3T3-L1 cells	Activited AMPK and SIRT1 pathways	↑ UCP1, PGC-1α, PPARα	[48]
Rose hip	Genus *Rosa*	DIO mice	Increased phosphoryl- ation of AMPK	↑ UCP1, Cidea, AMPK	[49]
Strawberry methanolic extract	*Fragaria x ananassa* cv. Romina	3T3-L1 cells	Upregulate PGC1α and p-AMPKα	↑ UCP1, PGC-1α, AMPKα, SIRT1 ↓ PPAR-γ	[50]
Protein	Milk	DIO mice	Activated AMPKα–PGC-1α–UCP1 axis	↑ UCP1, PGC-1α, AMPKα, Cidea	[51]
Acid-hydrolyzed silk peptide	*Bombyx mori* cocoons	DIO mice, primary sWAT cells	Upregulating AMPK phosphorylation	↑ UPC1, Prdm16, PGC-1α, UCP3 ↓ C/EBPα, FABP4	[52]
Resveratrol	*Veratrum grandiflorum*	DIO mice, stromal vascular fraction cells	Activated AMPKα1	↑ UCP1, Prdm16, PGC-1α	[53]
Seabuckthorn powder	*Hippophae rhamnoides* L.	DIO mice	Activated of AMPK/SIRT1 Pathway	↑ UCP1, Prdm16, PGC-1α, AMPK, SIRT1	[54]
Sea buckthorn pomace	*Hippophae rhamnoides* L.	ram lambs	Activated the AMPK–PGC-1α–UCP1 signaling pathway	↑ UCP1, PGC-1α, Prdm16	[55]
Luteolin	Fruits, vegetables	DIO mice	Activated AMPK/PGC1α signaling	↑ UCP1, PGC-1α	[56]
Resveratrol	*Veratrum grandiflorum*	DIO mice	Activated AMPK/Sirt1 pathway	↑ UCP1, Prdm16, PGC-1α, p-AMPKα, SIRT1	[54]

Arrows indicate direction of change compared to control: ↑, increase; ↓, decrease.

**Table 2:** Promotion of WAT browning via PPARs by dietary ingredients.

Compound Name	Source	Experimental Model	Mechanisms	Efficacy	Reference
Docosahexaenoic	Dietary	DIO mice, 3T3-L1 cells	Upregulated the PPARγ expression	↑ UCP1, Prdm16	[57]
Omega-3 fatty acids	*Salvia hispanica* L.	DIO mice	Upregulated the PPARγ expression	↑ UCP1, PGC-1α, PPARγ	[58]
Cucurbitacin B-, E-, and I	Melons	3T3-L1 cells	Possibly inhibited PPARγ	↑ UCP1, PGC-1a, Prdm16	[21]
Alcohol	Alcohol	DIO mice	Upregulated the expression of the PGC1-α/PPAR-α pathway protein and the P38 MAPK/CREB pathway	↑ UCP1, CXCL1, PPARα, SIRT1	[31]
Green tea catechins	Green tea	DIO rats	Through modulation of PPAR pathway	↑ UCP1, PPARγ, PPARδ, AOX	[59]
Lycopene	Tomato	3T3-L1 cells, primary adipocytes	Partly through induction of PPARγ activation.	↑ UCP1, PPARγ, Prdm-16, PGC-1α	[60]
Green tea extract	Green tea	DIO rats	Upregulation of PPARγ in adipocytes	↑ UCP1, PGC-1α, PPAR-γ, Prdm16, BMP-7, FGF-21, CPT-1, CIDEA	[32]
α-Monoglucosyl Hesperidin	Citrus peel	DIO mice	Activated PPARγ signaling	↑ UCP1	[61]
DHA	Fish oils	DIO mice, 3T3-L1 cells	Possibly activited DHA/GPR120/PPARγ signaling	↑ UCP1, Prdm16, PGC-1 α, TFAM,	[48]
Citrate	Ultra-processed foods	DIO mice	Activited PPARα	↑ UCP1, PGC-1α, PPARα; ↓ PPARγ	[33]
Monoterpene	Thyme	3T3-L1 cells	Activated PPARγ	↑ UCP1, PPARγ, PPARδ, HSL, PGC-1α	[62]
Goat’s milk	Goat’s milk	DIO mice	Activated PPARγ2	↑ UCP1, HSL	[34]
Purple Sweet Potato Extracts	Ipomoea batatas	DIO mice, 3T3-L1 cells	Upregulated the PPARγ expression	↑ PGC1a, UCP1, PPARγ	[52]
Artepillin C	Brazilian propolis	C3H10T1/2 cells, stromal vascular fraction cells, Normal diet mice	Activated PPARγ	↑ UCP1, Prdm16, Cidea, Elovl3	[63]
Palmitoyl lactic acid	Krill oil	3T3-L1 cells	Activated PPARγ	↑ UCP1, Prdm16, PGC-1α, PPARγ	[64]
Bitter Melon Seed Oil	Bitter Melon Seed	wild-type mice, PPARα-null male mice	Activated PPARα	↑ UCP1, PPARα, PPARγ	[65]

Arrows indicate direction of change compared to control: ↑, increase; ↓, decrease.

**Table 3:** Promotion of WAT browning via TRP channels by dietary compounds.

Compound Name	Source	Experimental Model	Mechanisms	Efficacy	Reference
Hydroxy-α-sanshool	*Zanthoxylum bungeanum Maxim*	DIO mice, 3T3-L1 cells	Associated with SIRT1-dependent PPAR-γ deacetylation through activating the TRPV1/AMPK pathway	↑ UCP1	[19]
Capsaicin	*Capsicum annuum*	DIO mice, wild-type, TRPV1 KO mice	Activited TRPV1 channels	↑ UCP1	[70]
Royal Jelly Tocotrienol	Nurse honeybees Vitamin E	DIO rats	Activited the TRP-SNS-UCP1 axis	↑ UCP1, Prdm16, CREB1, P38MAPK,	[71]
Menthol	*Mentha haplocalyx*	DIO mice, 3T3-L1 cells	TRPM8 mediated glucagon dependent mechanism of energy expenditure	↑ UCP1, PGC-1α, Prdm16, CIDA, FOXC2, TBX1, CD137	[72]
Capsaicin	*Capsicum annuum*	3T3-L1 cells	Activited TRPV1	↑ UCP1, SIRT1, Prdm16, Pgc1α, MAPK14; ↓ PPARγ	[73]
10-oxo-12(Z)-octadecenoic acid	Linoleic acid	DIO mice, KK-Ay mic, TRPV1-/-mice, HEK293 cells	Activated TRPV1	↑ UCP1, PGC-1α, β3-AR, Cpt1b, Prdm16, Tbx1	[74]

Arrows indicate direction of change compared to control: ↑, increase; ↓, decrease.

**Table 4:** Promotion of adipose tissue browning via β3-AR by diet or active ingredients.

Compound Name	Source	Experimental Model	Mechanisms	Efficacy	Reference
Trans-Cinnamic Acid	*Daemonorops draco* Bl.	3T3-L1 cells, HIB1B preadipocytes	Activated of the β3-AR and AMPK Signaling Pathways	↑ UCP1, Prdm16, PGC-1α, CD137, Cidea, Cited1, Tbx1, Tmem26, Lhx8, Ppargc1, Zic1; ↓ C/EBPα, PPARγ	[82]
D-mannitol	Algae, onions, grasses	3T3-L1 cells	Activated the β3-adrenergic receptor–PKA axis	↑ UCP1, Prdm16, PGC-1α, ACOX1, Cidea, CPT1, HSL, Tmem26, FGF21	[83]
Curcumin	Turmeric	Normal diet mice	Activated NE/β3-AR	↑ UCP1, PGC-1α, Prdm16, TMEM26, NE, β3-AR	[84]
Ethanol extracts from lotus leaf	*Nelumbo nucifera* Gaert	C3H10T1/2 mesenchymal stem cells	Possibly activited the β3-AR/AMPK signaling pathway	↑ UCP1, Sirt1, PGC-1α, Cidea, ATGL, HSL	[85]

Arrows indicate direction of change compared to control: ↑, increase; ↓, decrease.

**Table 5:** Promoting WAT browning via SIRTs by dietary components.

Compound Name	Source	Experimental Model	Mechanisms	Efficacy	Reference
Guttiferone	*Garcinia cambogia*	C3H10T1/2 cells, 3T3-L1 cells	Through boosting SIRT3-mediated browning	↑ UCP1, SIRT3; ↓ PPARγ	[88]
Sulforaphane	Broccoli	3T3-L1 cells	The upregulation of nuclear factor E2-related factor 2/sirtuin1/peroxisome proliferator activated receptor gamma coactivator 1 alpha signaling	↑ UCP1	[89]
Epicatechin	Green Tea	DIO mice, Human adipocytes	SIRT1 mediated PPAR γ Deacetylation activation	↑ UCP1, Prdm16, UCP2, irisin, PGC-1α, NRF1/2, DIO2, SIRT3	[90]
Apigenin	*Daphne genkwa*	DIO mice	Activated lipolysis (ATGL/FOXO1/SIRT1)	↑ UCP1, PGC-1α, ATGL, HSL, SIRT1, p-AMPK; ↓ NF-κB, MAPK	[91]
Resveratrol Oxyresveratrol	*Veratrum grandiflorum*	DIO mice	Activated Sirt1/PGC-1α pathway	↑ UCP1, Prdm16	[92]

Arrows indicate direction of change compared to control: ↑, increase; ↓, decrease.

**Table 6:** Diet or active ingredients that promote WAT browning via FGF21.

Compound Name	Source	Experimental Model	Mechanisms	Efficacy	Reference
Apple polyphenols	Apple	DIO mice	Possibly via activation/induction of the peripheral catecholamine synthesis-FGF21-PGC-1α cascade	↑ UCP1, Cidea, Tbx1, Cd137, FGF21, PGC-1α, Fgf21	[102]
Leucine Isoleucine	Dietary	DIO mice	Activated FGF21/SIRT1/PRDM16 pathway	↑ UCP1, Atgl, HSL, Tmem26, CD137, Cidea, Prdm16, SIRT1, FGF21	[103]
Maqui	*Aristotelia chilensis*	DIO mice	The improvement of FGF21 signaling	↑ UCP1, Prdm16, PGC-1α, PPARγ	[104]
Fish Oil	Fish	DIO mice	Possibly increased Fgf21	↑ UCP1, FGF21, β3-AR, PPARγ, Cidea	[35]

Arrows indicate direction of change compared to control: ↑, increase.

## Data Availability

No new data were created or analyzed in this study.

## Abbreviations

AT, adipose tissue; WAT, white adipose tissue; BAT, brown adipose tissue; UCP1, uncoupling protein 1; Prdm16, positive regulatory domain containing 16; ATP, adenosine triphosphate; AMPK, adenosine 5′-monophosphate-activated protein kinase; tCA, trans-cinnamic acid; SIRT1, silent in-formation regulator 1; PGC-1α, PPARγ 1 coactivator α; DIO, diet-induced obese; SDG, secoisolariciresinol diglucoside; β3-AR, β3-adrenergic receptor; Sal A, salvianolic acid A; HFD, high-fat diet; siRNA, small interfering RNA; UPF, ultra-processed foods; PMQ, pentamethylquercetin; PKA, protein kinase A; Leu, leucine; Ile, isoleucine; HAS, hydroxy-α-sanshool; PPARγ, peroxisome proliferator-activated receptor-γ; PPARγ2, peroxisome proliferator-activated receptor-γ2; T3, triiodothyronine; PPARα, peroxisome proliferator-activated receptor-α; ACC, acetyl-CoA carboxylase; C/EBPα, CCAAT/enhancer binding protein α; UCP3, uncoupling protein 3; TRPV1, transient receptor potential vanilloid type-1; Tbx1, T-box transcription factor; TRP, transient receptor potential; Tmem26, transmembrane protein 26; UCP2, uncoupling protein 2; TRPM8, transient receptor potential cation channel subfamily melastatin member 8; SIRT3, silent in-formation regulator 3; FGF21, fibroblast growth factor 21; cGMP, cyclic guanosine monophosphate; Cidea, cell death-inducing DFFA-like effector a; HSL, hormone sensitive lipase; IngSAT, inguinal subcutaneous adipose tissue; TCA, tricarboxylic acid; HDEA, 10-hydroxy-Trans-2-decenoic acid; DHA, docosahexaenoic acid.
